# The carbon isotopic signature of C_4_ crops and its applicability in breeding for climate resilience

**DOI:** 10.1007/s00122-020-03761-3

**Published:** 2021-02-11

**Authors:** Stella Eggels, Sonja Blankenagel, Chris-Carolin Schön, Viktoriya Avramova

**Affiliations:** Plant Breeding, TUM School of Life Sciences, Technical University of Munich, Liesel-Beckmann-Straße 2, 85354 Freising, Germany

## Abstract

**Key message:**

**Carbon isotope discrimination is a promising trait for indirect screening for improved water use efficiency of C**_**4**_** crops**.

**Abstract:**

In the context of a changing climate, drought is one of the major factors limiting plant growth and yield. Hence, breeding efforts are directed toward improving water use efficiency (WUE) as a key factor in climate resilience and sustainability of crop production. As WUE is a complex trait and its evaluation is rather resource consuming, proxy traits, which are easier to screen and reliably reflect variation in WUE, are needed. In C_3_ crops, a trait established to be indicative for WUE is the carbon isotopic composition (δ^13^C) of plant material, which reflects the preferential assimilation of the lighter carbon isotope ^12^C over ^13^C during photosynthesis. In C_4_ crops, carbon fixation is more complex and δ^13^C thus depends on many more factors than in C_3_ crops. Recent physiological and genetic studies indicate a correlation between δ^13^C and WUE also in C_4_ crops, as well as a colocalization of quantitative trait loci for the two traits. Moreover, significant intraspecific variation as well as a medium to high heritability of δ^13^C has been shown in some of the main C_4_ crops, such as maize, sorghum and sugarcane, indicating its potential for indirect selection and breeding. Further research on physiological, genetic and environmental components influencing δ^13^C is needed to support its application in improving WUE and making C_4_ crops resilient to climate change.

## Improved water use efficiency to mitigate for the effect of changing climatic conditions

Climate change comprises a variety of environmental changes, including increases in CO_2_ concentrations, temperatures and unstable precipitation (Hatfield and Dold 2019). Since these environmental factors have a strong influence on key plant processes, affecting both photosynthesis and water relations, plant performance needs to be optimized under new climatic conditions and limitations. Water deficit is one of the major factors impairing crop growth and yield (Leakey et al. 2019). Therefore, a main focus of improving the resilience of plants to the changing climatic conditions is increasing their water use efficiency (WUE) to enhance sustainability of agriculture, save water and contribute to food security (Condon et al. 2004; Leakey et al. 2019).

**Fig. 1:**
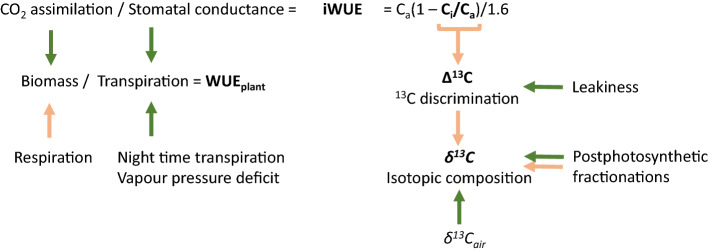
Associations between water use efficiency (WUE) and the carbon isotopic composition of C_4_ plant material. Negative effects are depicted by light orange arrows, positive effects are depicted by dark green arrows. The WUE of a plant (WUE_plant_) can be assessed by the destructive measurement of biomass in relation to the sum of water transpired by the plant. The biomass, which the plant accumulates, depends on assimilation rate and respiration, while the water transpired by the plant depends on the stomatal conductance, as well as night time transpiration and the vapor pressure deficit of the air over its lifetime. The intrinsic WUE (iWUE) is defined as the ratio of assimilation rate over stomatal conductance of a leaf section at a specific time and is by definition related to the ratio of the intercellular CO_2_ concentration (C_i_) to the ambient CO_2_ concentration (C_a_; Yang et al. 2016). This ratio of C_i_/C_a_ is theoretically negatively correlated to the discrimination against the ^13^C isotope during assimilation (∆^13^C), when the influence of leakiness is stable below 0.37 as it was observed, e.g., in Henderson et al. (1992). The isotopic composition of tissues like leaves and grains (δ^13^C) is an indirect and integrated measure for ∆^13^C, when the isotopic composition of the air (δ^13^C_air_) is accounted for. Post-photosynthetic fractionations influence δ^13^C further as these fractionations lead to distinct isotopic signatures of different plant compounds, which through their relative contribution to the composition of a tissue determine its δ^13^C

In the context of plant production, WUE is defined as the ratio of yield (grain or biomass) to water received or evapotranspired by the system (e.g., field plot, Ellsworth and Cousins 2016). In a more narrow sense, WUE at the single plant level (WUE_plant_) represents the amount of biomass produced per volume of water transpired. The main component of WUE_plant_ is the intrinsic WUE (iWUE) at the leaf level, representing the ratio of CO_2_ assimilation rate to stomatal conductance (Fig. 1, Medrano et al. 2015). As both CO_2_ assimilation and stomatal conductance are influenced by several environmental and genetic factors, iWUE is a complex trait. In addition to iWUE, important components of WUE_plant_ are the air water vapor pressure deficit, which is the difference between the amount of moisture in the air and the maximum air moisture at saturation, nighttime transpiration and carbon loss through respiration (Ellsworth et al. 2020). A high iWUE can either be achieved through an increase in CO_2_ assimilation rate without a corresponding increase in stomatal conductance or by reducing stomatal conductance without a corresponding decrease in CO_2_ assimilation rate (Leakey et al. 2019). In C_4_ crops, high assimilation rates can be realized at relatively low stomatal conductance due to the specific characteristics of the C_4_ cycle and its CO_2_ concentrating mechanism, leading to an elevated iWUE (Way et al. 2014). In severe drought, however, it has been shown, that the advantage in WUE of C_4_ grasses, such as maize (*Zea mays* L.), disappears (Blankenagel et al. 2018). Several studies have demonstrated significant genetic variation for WUE within C_4_ species and thus potential for further genetic improvement of WUE (Geetika et al. 2019; Hammer et al. 1997; Henderson et al. 1998; Jackson et al. 2016; Leakey et al. 2019; Ryan et al. 2016; Sinclair 2012; Xin et al. 2009).

Despite increased efforts focused on improving WUE, the complexity of the trait has restricted the breeding progress in this area (Chen et al. 2011). The high number of physiological factors determining WUE and the variability of the plants’ responses to different environments have impeded the use of traditional breeding methods directed at WUE improvement (Leakey et al. 2019). Additionally, the difficulty of high-throughput screening for WUE has been a major limiting factor. Screening of WUE_plant_ requires gravimetric tracking of water uptake and destructive measurement of biomass production. Screening for iWUE with gas exchange measuring systems is also very time- and labor-intensive. Therefore, both WUE_plant_ and iWUE are difficult to measure on large populations needed for successful breeding (Chen et al. 2011). Additionally, screening of WUE is typically performed in phenotyping platforms with controlled environmental conditions (Ryan et al. 2016) and it has often been difficult to translate the results to the performance under field conditions (Araus and Cairns 2014). Hence, the identification of proxy traits that are easy to measure on a large number of plants, and reliably reflect variation in WUE would greatly support advances in breeding for drought resistance (Chen et al. 2011; Leakey et al. 2019). In C_3_ plants, such a proxy trait is carbon isotope discrimination (Δ^13^C), which describes the preferential assimilation of the lighter carbon isotope ^12^C over the heavier ^13^C during the process of photosynthesis. The extent of this discrimination is dependent on the ratio of intercellular to ambient CO_2_ partial pressure (C_i_/C_a_), determined by CO_2_ assimilation rate and stomatal conductance. Since this dependence is shared with WUE, Δ^13^C is reflective of environmental conditions affecting CO_2_ assimilation, stomatal conductance and genotypic differences in WUE. When plants are grown under uniform environmental conditions, Δ^13^C has been established to be indicative for genotypic differences in WUE as well as yield under drought (Farquhar and Richards 1984; Saranga et al. 1998). Therefore, Δ^13^C has been applied in a breeding program and giving rise to more water use efficient wheat varieties (Condon et al. 2004).

For C_4_ species, the use of Δ^13^C as a proxy for WUE is less clear due to the more complex nature of carbon fixation and Δ^13^C compared to C_3_ species (Farquhar 1983). In addition to the ratio of CO_2_ assimilation rate and stomatal conductance, the leakage of CO_2_ from the bundle sheath cells back to the mesophyll determines Δ^13^C as an additional contributing factor (Fig. 1, Farquhar 1983). This leakage is affected by the coordination of different photosynthetic enzymes and influences the efficiency of photosynthesis. Therefore, in addition to studies focused on WUE, Δ^13^C is of high interest for studying limitations of photosynthetic efficiency, especially in response to changing environmental conditions (Kromdijk et al. 2014). Due to the difficulties of integrating all the abovementioned components, Δ^13^C research in C_4_ crops has not advanced as actively as in C_3_ plants. Only recently, due to the progress in phenotyping and genotyping technologies, there have been advances in our understanding of the factors influencing both Δ^13^C and WUE as well as their interconnectivity in C_4_ plants. For broadening our knowledge in this research area, the combination of genetic studies, identifying underlying quantitative trait loci (QTL) and the universality of their effects in different genetic backgrounds and environments, with physiological studies, unraveling the interaction of different Δ^13^C determinants and their environmental dependence, is needed.

This review will provide an overview of the current knowledge on carbon isotope discrimination in C_4_ plants in the context of breeding for enhanced water use efficiency.

## Carbon isotope discrimination during carbon assimilation and its theoretical connection to WUE

Carbon naturally occurs as two stable isotopes, ^12^C and ^13^C, the latter of which is only present in 1.1% of CO_2_ in the atmosphere (Farquhar et al. 1989a). In plants, the ^13^C/^12^C ratio is even lower than in air, indicating that plants discriminate against the heavier isotope. This discrimination happens mainly during photosynthetic CO_2_ assimilation by the plant. The stable carbon isotopic composition of a sample, e.g. air or plant material, (δ^13^C) is conventionally expressed as the ^13^C/^12^C ratio of the sample (R_s_) in reference to the ^13^C/^12^C ratio of the Pee Dee Belemnite Standard (R_PDB_), a fossil with an exceptionally high amount of the ^13^C isotope (Eq. (1), Farquhar et al. 1982).

1 $$\delta^{13} C \, = \, R_{s} / \, R_{\text{PDB}} - \, 1$$

This results in current values for δ^13^C in the air (δ^13^C_air_) of about − 8.5  ‰, with a trend to decrease over the years due to the increase in anthropogenic emissions (Graven et al. 2017). The difference between the δ^13^C of the analyzed plant sample (δ^13^C_p_; typically plant dry matter) and δ^13^C_air_ surrounding the plant is described by the carbon isotope discrimination (Δ^13^C) of plants (Eq. (2), Farquhar et al. 1982).

2 $$\Delta^{13} C = \, (\delta^{13} C_{\text{air}} - \delta^{13} C_{p} ) \, (1 + \delta^{13} C_{\text{air}} )^{ - 1}$$

Due to the discrimination against ^13^C during carbon assimilation, δ^13^C of plant material shows more negative values than that of air. The average δ^13^C of C_3_ plant tissue is around − 28  ‰, corresponding to a Δ^13^C of 20  ‰ (Farquhar et al. 1989a). Δ^13^C during C_3_ photosynthesis is characterized primarily by the more frequent use of the ^12^C over ^13^C isotope by Rubisco (Ribulose-1,5-bisphosphate carboxylase/oxygenase), the main enzyme contributing to carbon fixation, owing to a lower reactivity of ^13^C. Additionally, several alterations in the ^13^C/^12^C ratio of CO_2_, called isotopic fractionations, occur during diffusion of CO_2_ from the atmosphere to the site of carbon fixation (Farquhar et al. 1982). Since these fractionation factors of Rubisco carboxylation and diffusion are relatively constant, in C_3_ plants a linear positive correlation between Δ^13^C and the ratio of intercellular (C_i_) to ambient (C_a_) CO_2_ partial pressure (C_i_/C_a_) is predicted and observed (Farquhar et al. 1989a). C_i_/C_a_, on the other hand, is determined mainly by stomatal conductance and photosynthetic capacity and thus directly connected to intrinsic water use efficiency. As a consequence, a strong inverse correlation between Δ^13^C and WUE can be expected in C_3_ plants. Several studies on a variety of C_3_ species, including important agricultural crops like wheat, barley, soybean, peanut, cotton, rice, potato and tomato, have confirmed this inverse relationship between WUE and Δ^13^C experimentally using both dry matter derived estimates (Barbour et al. 2010; Condon et al. 2004; Hubick and Farquhar 1989; Hubick et al. 1986; Impa et al. 2005; Martin et al. 1999; Saranga et al. 1998; Vos and Groenwold 1989) and short-term measurements (Evans et al. 1986) of Δ^13^C. These analyses build the foundation for the application of Δ^13^C in breeding for improved WUE of C_3_ plants.

**Fig. 2:**
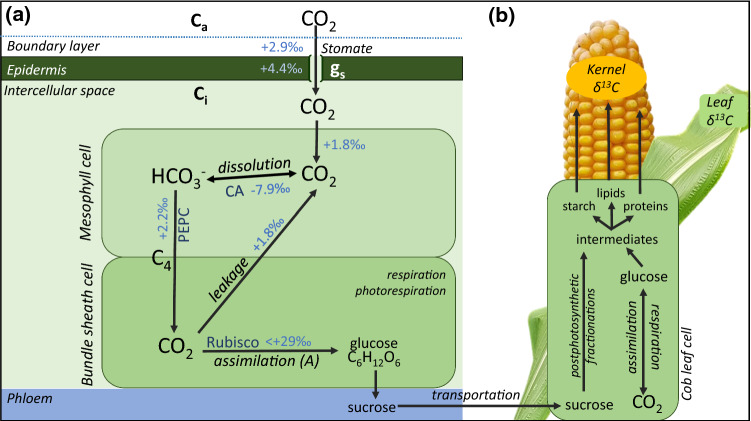
Simplified presentation of the factors influencing carbon isotope discrimination (**a**) and the resulting isotopic composition (δ^13^C) of leaves and grains (**b**) in C_4_ plants. **a** CO_2_ entering the leaf diffuses through the boundary layer and stomata (stomatal conductance g_s_), whereby discriminations against the ^13^C isotope (discrimination factors are shown in lighter blue and were reviewed by Ubierna et al. (2018b)) take place. Diffusion in the cytoplasm of mesophyll cells contributes further to discrimination against ^13^C, whereas there is an enrichment in ^13^C accompanying the conversion of CO_2_ to HCO_3_^−^, catalyzed by carbonic anhydrase (CA) and a relatively small discrimination during fixation by phosphoenolpyruvate carboxylase (PEPC). By active transportation via C_4_ dicarboxylic acids (malate or aspartate), CO_2_ is enriched in the bundle sheath cell. The discrimination realized by Ribulose-1,5-bisphosphate carboxylase/-oxygenase (Rubisco) depends on the leakage of CO_2_ back into the mesophyll, which itself comes with a discrimination factor. Additional factors influencing the discrimination during assimilation are respiration and photorespiration. For more details we refer the reader to an excellent review by von Caemmerer et al. (2014). The ratio between intercellular CO_2_ concentration (C_i_) and ambient CO_2_ concentration (C_a_), which determines the intrinsic water use efficiency, is correlated with the ^13^C discrimination. **b)** Assimilates, carrying an isotopic signature influenced by C_i_/C_a_ during their assimilation can be transported, predominantly as sucrose, via the phloem and unloaded in sink tissues, where they contribute to the carbon isotopic composition of these tissues (δ^13^C). Additionally, the glucose assimilated in the tissue itself and other compounds like starch, lipids and proteins determine δ^13^C. Due to post-photosynthetic fractionations during their synthesis, starch, lipids and proteins carry distinct isotopic signatures. The relative composition of compounds of distinct isotopic signatures is likely to contribute to differences observed when measuring the isotopic composition in whole tissues of leaves and grains (grain δ^13^C, leaf δ^13^C)

In C_4_ species, the carbon concentrating mechanism is determined by the Kranz anatomy, locally separating the initial carbon fixation from the Rubisco-catalyzed CO_2_ assimilation in mesophyll and bundle sheath cells, respectively. This, in turn, leads to additional complexity of Δ^13^C (Farquhar 1983; von Caemmerer et al. 2014; Fig. 2). A comprehensive model of Δ^13^C of C_4_ plants is described by Farquhar and Cernusak (2012). A more simplified model of Δ^13^C as a function of leakiness (φ) and C_i_/C_a_ is given by Eq. (3) (Farquhar 1983).

3 $$\Delta^{13} C \, = \, a \, + \, (b_{4} + \, \left( {b_{3} - \, s} \right)\varphi - \, a) \, C_{i} C_{a}^{ - 1}$$

Values for the fractionation factors, including the fractionation during diffusion of CO_2_ in air (a), in the liquid phase (s), Rubisco carboxylation (b_3_) and the combined fractionation of CO_2_ dissolution and PEPC carboxylation (b_4_) are reviewed by Ubierna et al. (2018b). After diffusion through the stomata (a = 4.4‰), CO_2_ is converted to bicarbonate by carbonic anhydrase in the mesophyll cells and subsequently fixed by the phosphoenolpyruvate carboxylase (PEPC). Due to fractionation during dissolution of CO_2_, the bicarbonate is enriched in ^13^C. Since the discrimination against ^13^C by PEPC is smaller than the enrichment during dissolution, there is an overall ^13^C enrichment during this initial fixation step to a C_4_ acid (b_4_ ≈ − 5.7  ‰, Farquhar 1983). The C_4_ acid is then transported to the bundle sheath cell and decarboxylated. The released CO_2_ is re-fixed by Rubisco. Here, the discrimination by Rubisco (b_3_ ≈ 29‰) depends on the leakage of some CO_2_ back to the mesophyll cell (Farquhar 1983). This leakage originates from the concentration gradient between the two cell types and is quantified by the leakiness, defined as the fraction of CO_2_ previously fixed by PEPC that leaks back to the mesophyll cells. Values for leakiness can theoretically range from 0 to 1 and depend on the CO_2_ gradient between the two cell types, determined by the ratio of PEPC and Rubisco carboxylation rates, as well as on the conductance of bundle sheath cells (Henderson et al. 1992; von Caemmerer and Furbank 2003). A higher leakiness enables a higher discrimination by Rubisco, since it allows some ^13^C to be released from the bundle sheath cell. Additionally, some fractionation occurs during leakage itself (s = 1.8  ‰, Henderson et al. 1992). Overall, due to the ^13^C enrichment in the initial fixation step and due to the dampened Rubisco discrimination, caused by the restricted CO_2_ release from the bundle sheath cell, Δ^13^C is lower in C_4_ plants with values typically around 4–8 ‰ (Farquhar 1983; Henderson et al. 1992; von Caemmerer et al. 2014) as compared to 16–21 ‰ in C_3_ plants (Kohn 2010; O’Leary 1988). In C_4_ plants, it has also been described, that variation of Δ^13^C accompanying changes in C_i_/C_a_ is smaller compared to C_3_ plants (Evans et al. 1986; Henderson et al. 1992, 1998). Depending on leakiness, the relationship between Δ^13^C and C_i_/C_a_ and consequently WUE can theoretically be positive, negative or zero, with zero correlation at a leakiness of 0.37 (Farquhar et al. 1989a). Estimates of leakiness in experiments on a variety of C_4_ species using simultaneous measurements of on-line Δ^13^C and gas exchange have been reported to be lower than 0.3, leading to a positive relationship between Δ^13^C and WUE (Ellsworth and Cousins 2016).

In summary, compared to C_3_ plants a weaker correlation between Δ^13^C and WUE can be expected in C_4_ plants. If bundle sheath leakiness is relatively constant, as suggested by experimental values for sorghum (*Sorghum bicolor*) and *Amaranthus edulis* (Henderson et al. 1992, 1998; Sonawane and Cousins 2020), it should be possible to use Δ^13^C as a proxy trait in breeding for developing more water use efficient C_4_ crops.

## Methods to assess carbon isotope discrimination (Δ^13^C)

Measurements of Δ^13^C values require sensitive and well-standardized methods to reduce environmental influences and temporal changes. To be applied in breeding, it is additionally important that measurements are not excessively time- and labor-intensive and can be assessed at an early developmental stage.

Short-term measurements of Δ^13^C, assessing the concurrent change in δ^13^C in the air entering and exiting a leaf cuvette, can be performed with a continuous flow isotope ratio mass spectrometer (CF-IRMS) combined with an infrared gas analyzer (Kubasek et al. 2007). These on-line measurements of Δ^13^C give a direct measure of the photosynthetic discrimination, allow to follow short-term changes in response to changing environmental conditions and can be used to study different components of the C_4_ pathway, including leakiness and mesophyll conductance (von Caemmerer et al. 2014). For the purpose of screening for WUE, these measurements are not suitable, since on-line IRMS measurements are more time-consuming and of higher cost than measuring the direct trait (Cernusak et al. 2013). An alternative to on-line measurements of Δ^13^C by IRMS are measurements by tunable diode laser absorption spectroscopy (TDLAS) which allow higher throughput, offer application in the field and come at lower cost. For these reasons, they have been used more frequently in Δ^13^C research in recent years (Ubierna et al. 2018b). The precision of TDLAS for CO_2_ isotopologues is reported to be 0.2 ‰ compared with ≤ 0.1 ‰ for IRMS (Cui et al. 2018). Therefore, TDLAS might be less potent to detect the small differences in ∆^13^C of C_4_ plants (Table 1). While short-term measurements of gas exchange give a direct reflection of the current photosynthetic processes, they are sensitive to environmental and developmental fluctuations (De Souza et al. 2018; Medrano et al. 2015) as well as to time and day of measurement, as gas exchange and ∆^13^C follow diurnal cycles (Matthews et al. 2017; Niu et al. 2003; Stangl et al. 2019).

**Table 1:** Intraspecific variation of carbon isotope discrimination/composition in C_4_ plants

Species	Genetic material	Carbon isotopic composition (δ^13^C) in ‰	Carbon isotope discrimination (∆^13^C) in ‰	Maximum genotypic difference (‰)	Tissue	References
*Zea mays*	50 commercial inbred lines	− 11.6 to − 10.7		0.9	Grain	Tieszen and Fagre (1993)
	59 diverse accessions	− 12.00 to − 9.86		2.14	Grain	
	193 diverse accessions	− 11.5 to − 9.7		1.8	Grain	
	6 lines with contrasting drought tolerance, 35 hybrids, 2 drought tolerant and 2 drought sensitive inbred lines		4.88 to 5.41	0.53	Leaf	Monneveux et al. (2007)
			4.10 to 4.54	0.44	Ears	
	16 hybrids, one commercial hybrid as a check		4.98 to 5.53	0.55	Leaf	Cabrera-Bosquet et al. (2009)
			3.59 to 4.01	0.42	Grain	
	Mean of 15 tropical inbred lines and mean of 16 of their hybrids		5.30 to 5.64	0.34	Leaf	Araus et al. (2010)
			3.82 to 4.01	0.19	Grain	
	2 varieties	− 14.78 to − 13.13		1.65	Leaf	Pengelly et al. (2011)
		− 15.08 to − 15.02		0.06	Husk	
	89 introgression lines, derived from a dent and a flint inbred line		4.24 to 5.84	1.6	Grain (field)	Gresset et al. (2014)
			4.98 to 6.55	1.57	Grain (GH)	
			5.42 to 6.98	1.56	Leaf (GH)	Gresset (2014)
	29 inbred lines (including 26 NAM^a^ founders)	− 15.0 to − 13.7		1.3	Leaf	Kolbe et al. (2018)
	31 inbred lines (including 26 NAM founders)	− 13.02 to − 11.61 (2015)		1.41	Leaf	Twohey et al. (2019)
		− 13.29 to − 12.22 (2016)		1.07	Leaf	
*Panicum coloratum*	4 varieties	− 12.74 to − 11.36		1.38	Leaf	Ohsugi et al. (1988)
*Saccharum spp.* Hybrid	2 cultivars		4.4 to 4.7	0.3	Leaf	Meinzer et al. (1994)
	4 cultivars		3.2 to 3.9	0.7	Leaf	Saliendra et al. (1996)
*Sorghum bicolor* Moench	12 genotypes		4.24 to 4.84	0.6	Leaf	Hubick et al. (1990)
	45 cultivars		3.10 to 4.15	1.05	Leaf	Hammer et al. (1997)
	30 lines		2.46 to 2.89	0.43	Leaf (GH)	Henderson et al. (1998)
	4 lines		3.43 to 4.10	0.67	Leaf (field)	

^a^NAM, nested association mapping, the NAM founder lines include the 26 most extensively researched maize lines, which represent a broad cross section of modern maize diversity (Yu et al. 2008)

GH, greenhouse

Alternatively, Δ^13^C can be estimated from δ^13^C of plant dry matter or extracted plant compounds (e.g., photosynthetic assimilates such as sugars) measured by IRMS. Differences in the δ^13^C of genotypes evaluated in the same experiment reflect variation in Δ^13^C, because it can be assumed that δ^13^C_air_ was the same for all plants. For comparison across experiments, δ^13^C_air_ has to be known or assessed to derive Δ^13^C (see Eq. (2)). While assessing δ^13^C of plant material requires destructive sampling, it has the advantage of being independent of a measurement time point. Thus, these measurements are less affected by errors due to external factors and allow high numbers of samples to be screened. Since the photosynthetic assimilates are used for plant syntheses, dry matter δ^13^C of, e.g., leaves or grains is assumed to be a time-integrated measure of Δ^13^C over the period of tissue growth (Ellsworth and Cousins 2016; Pate 2001). By integrating the diurnal, developmental and environmental fluctuations in C_i_/C_a_ that would also affect WUE_plant_, dry matter δ^13^C has an additional advantage over on-line measurements, which can only reflect iWUE at the time point of measurement.

Differences between short-term measurements and dry matter derived estimates of Δ^13^C can also originate from post-photosynthetic fractionations (Henderson et al. 1992; Kubasek et al. 2007; von Caemmerer et al. 2014). Post-photosynthetic fractionations occur during metabolic reactions associated with the synthesis of different plant compounds (Hobbie and Werner 2004; Tcherkez et al. 2011) and during dark-respiration (Ghashghaie and Badeck 2014). Preferential export or incorporation of certain metabolite pools with distinct δ^13^C (Badeck et al. 2005, 2009; Bögelein et al. 2019) is hypothesized to then influence bulk leaf δ^13^C.

The sum of these additional fractionation processes can cause measurements of dry matter derived Δ^13^C to deviate from on-line Δ^13^C (Henderson et al. 1992; Kubasek et al. 2007) and can lead to weak or non-significant correlations of on-line and dry matter Δ^13^C over different C_4_ species of various C_4_-decarboxylation types as shown by Henderson et al. (1992) and Cousins et al. (2008). It is not established, whether post-photosynthetic fractionations significantly contribute to intraspecific variation of Δ^13^C in C_4_ plants and therefore affect the correlation of δ^13^C and WUE over different genotypes. The only study on this topic we are aware of was performed on diverse maize lines by Kolbe et al. (2018). Here, an RNA-sequencing approach did not reveal any indications for differences in post-photosynthetic metabolism that could be related to Δ^13^C differences between genotypes.

In the literature, next to leaves, grains are the most commonly sampled tissue, with absolute values for grain Δ^13^C being lower than for leaf Δ^13^C (Cabrera-Bosquet et al. 2009; Cernusak et al. 2009, Table 1). The correlation between the two measurements has been observed to be low in C_4_ species as well as in C_3_ species (Merah et al. 2001; Condon et al. 2004; Gresset 2014). On the one hand, these differences could originate from different temporal effects with leaf δ^13^C being more reflective of earlier vegetative growth and grain δ^13^C being more indicative of the conditions later in the growth period around flowering and grain filling (Condon et al. 2004; Cernusak et al. 2009). On the other hand, differences in biochemical composition or in the δ^13^C of sucrose exported for grain filling are likely to contribute to the disparity.

Overall, dry matter δ^13^C and the derived Δ^13^C are useful measures for screening for time-integrated WUE, if a reliable connection to WUE can be established. Dry matter δ^13^C is less measurement time sensitive than gas exchange measurements of iWUE and less destructive than WUE_plant_ measurements. For the tissue to be sampled, leaves are recommendable over grains, as they resemble more closely a time-integrated measure of iWUE and allow sampling during early developmental stages.

## Genetic analyses of Δ^13^C

Given that two of the main determinants of Δ^13^C, CO_2_ assimilation rate and stomatal conductance, are known to be complex polygenic traits in crops with C_3_ as well as C_4_ photosynthesis (Prado et al. 2018; van Bezouw et al. 2019), the genetic composition of Δ^13^C can be expected to be complex as well.

In C_3_ species, Δ^13^C has been shown to be determined by multiple QTL with small individual effects (Chen et al. 2011). In populations of C_3_ crops successfully used for QTL mapping, the intraspecific genetic variation for leaf or above-ground dry matter derived Δ^13^C was shown to be quantitative with maximal genotypic differences of about 1.2–2.3 ‰ in wheat (Rebetzke et al. 2008), 2.5 ‰ in soybean (Bazzer et al. 2020) and 3–4  ‰ in barley (Chen et al. 2012). Heritability was shown to be high for model plants such as *Arabidopsis thaliana* (0.67, Easlon et al. 2014) and crops such as wheat (0.63–0.86; Rebetzke et al. 2008). Regarding genotype by environment interactions (GxE) contrasting reports exist, but generally the genetic component seems to be much larger (Chen et al. 2011). In Arabidopsis, genes with pleiotropic effects on Δ^13^C, WUE and stomatal conductance have been identified (Des Marais et al. 2014; Franks et al. 2015; Masle et al. 2005; Nilson and Assmann 2010; Yang et al. 2016). Causal genes affecting Δ^13^C and WUE through effects on stomatal conductance were also identified in tomato (Bradford et al. 1983; Thompson et al. 2007) and potato (Antunes et al. 2012). Interactions of individual QTL for Δ^13^C with the genetic background have been demonstrated for several C_3_ crops, including soybean (Bazzer et al. 2020).

In C_4_ crop breeding, the use of Δ^13^C as an indirect selection criterion for improvement of WUE would require sufficient natural variation and a heritability comparable to C_3_ plants. Several studies have demonstrated that significant intraspecific variation for Δ^13^C exists, which might be indicative of differences in WUE and can be exploited for quantitative genetic studies to identify genomic regions controlling Δ^13^C. Evidence for significant intraspecific variation has been shown for maize, sorghum, sugarcane and *Panicum coloratum* (Table 1). For maize, several studies have explored variation in δ^13^C between different genotypes and results strongly depended on the investigated genetic material (Table 1). Significant genotypic differences were found mainly in sets with high genetic diversity and in material for which differences in drought tolerance and WUE were expected. For example, for δ^13^C in grain sampled from two diverse maize populations, a fairly large range of phenotypic values was observed (extremes differing by 2.1 ‰ and 1.8 ‰) as compared to a panel of inbred lines from a commercial breeding program with much less differentiation (0.9 ‰, Tieszen and Fagre 1993). Another example is the study of Monneveux et al. (2007), who found significant genotypic differences in leaf and ear δ^13^C between drought tolerant maize hybrids, drought tolerant inbred lines and susceptible inbred lines. Across drought tolerant hybrids, however, for which variation for WUE was likely reduced through previous selection for yield under drought, differences in Δ^13^C were small or absent. Similar results were shown in the study conducted by Cabrera-Bosquet et al. (2009), who studied maize hybrids derived from the same population with improved drought tolerance. Among a genetically diverse set of maize inbred lines frequently used in maize research (Gage et al. 2020), maximal genotypic differences of leaf δ^13^C were between 1.1 and 1.4 ‰ depending on the environment (Kolbe et al. 2018; Twohey et al. 2019) with a medium heritability of 0.57. High genotypic differences of up to 1.6 ‰ as well as a heritability of 0.69 have been demonstrated for grain δ^13^C in a maize introgression library derived from a drought tolerant dent recurrent parent and a drought susceptible flint donor parent (Avramova et al. 2019; Gresset et al. 2014). Thus, in the C_4_ species maize significant genotypic variation for δ^13^C exists, setting the stage for studies in C_4_ plants to investigate if δ^13^C could be predictive for WUE.

In recent years, several QTL studies for δ^13^C have been conducted in C_4_ plants. QTL for leaf δ^13^C explaining 6.5–14.5% of the genetic variance have been mapped in the C_4_ model grass *Setaria* (Ellsworth et al. 2020). In an interspecific recombinant inbred line (RIL) population of the two species *Setaria viridis* and *Setaria italica,* with a large phenotypic range for δ^13^C of 2.3 ‰, three QTL were identified with positive alleles contributed by both parents. Under drought-treatment with reduced variation for C_i_/C_a_ due to stomatal closure, on the other hand, no QTL could be detected.

In maize, using an introgression library, Gresset et al. (2014) identified 22 target regions with an effect on grain δ^13^C distributed over all 10 chromosomes. For 12 of the 22 regions the donor parent alleles affected δ^13^C positively, for the remaining 10 regions negatively. Of the identified QTL, one region explained 15% of the phenotypic variance and four others more than 5%, respectively. Absolute additive effects assigned to these regions were 0.20–0.31 ‰. A recent QTL analysis of leaf δ^13^C in maize was based on four RIL families derived from four different inbred lines crossed to B73 as the common parent (Sorgini et al. 2020). In this study, five QTL, which explained around 7–21% of the phenotypic variance were identified. Interestingly, three of these QTL overlap with QTL identified for grain δ^13^C by Gresset et al. (2014). This might indicate that the detected QTL for δ^13^C acted independently of the genetic background and points to a connection of leaf and grain δ^13^C. Contrarily, none of the leaf δ^13^C QTL were shared between the four different RIL families (Sorgini et al. 2020). As described for *Setaria* by Ellsworth et al. (2020), genotypic differences in maize δ^13^C were found to be reduced under low precipitation in the field (Avramova et al. 2019; Twohey et al. 2019). Hence, screening for δ^13^C is preferably to be performed under well-watered conditions to achieve better genetic differentiation of genotypes, which has also been concluded for C_3_ crops (Rebetzke et al. 2008).

There are contrasting reports regarding the relevance of GxE interactions for δ^13^C in C_4_ crops. In sorghum, Henderson et al. (1998) found indications for considerable GxE interaction between different field and greenhouse experiments. In maize, Twohey et al. (2019) detected changes in the ranking of genotypes regarding δ^13^C between field and greenhouse only for a few genotypes and for the maize introgression library described in Gresset et al. (2014) there was no significant GxE interaction.

In summary, although genetic analyses of δ^13^C in C_4_ crops are still scarce, existing studies point to the usefulness of δ^13^C for indirect selection for WUE, justified by significant genetic variation and medium to high heritability. Due to its relation with stomatal conductance, screening potential for WUE is higher in well-watered compared to water limited conditions.

## Correlation of δ^13^C and WUE in C_4_ plants

In addition to the requirement of significant genetic variation for both δ^13^C and WUE to select for more water use efficient plants, the central question to be resolved is whether a reliable correlation between δ^13^C and WUE exists in C_4_ species. In C_3_ crops, a positive correlation between δ^13^C and WUE is expected, because high C_i_/C_a_, corresponding to low WUE, allows for a high discrimination (Farquhar et al. 1989b). This relationship between δ^13^C and WUE has been shown at different levels, including correlation of on-line Δ^13^C and C_i_/C_a_ (Evans et al. 1986), correlation of leaf δ^13^C and WUE_plant_ (Farquhar and Richards 1984), correlation of leaf δ^13^C and yield under drought (Rebetzke et al. 2002) and colocalization of δ^13^C and WUE QTL (Adiredjo et al. 2014). In C_4_ plants, the correlation between δ^13^C and WUE could theoretically be positive or negative depending on leakiness. From the reported on-line measurements of Δ^13^C and C_i_/C_a_ with values of leakiness below 0.37, a negative correlation between δ^13^C and WUE would be expected (Farquhar et al. 1989a; Henderson et al. 1992), which is in contrast to the positive association in C_3_ plants. Consistent with theory, Twohey et al. (2019) found a negative correlation between leaf δ^13^C and WUE as well as positive association of δ^13^C and transpiration over three different watering regimes in an experiment including four maize RILs. Decreases of δ^13^C under water deficit, when stomatal closure decreases C_i_/C_a_ and increases WUE, have further been observed in several C_4_ species, including *Setaria* (Ellsworth et al. 2017), pearl millet (Brück et al. 2000), maize (Dercon et al. 2006), Australian C_4_ grasses (Ghannoum et al. 2002) and sorghum (Sonawane and Cousins 2020; Williams et al. 2001). These results indicate that changes in C_i_/C_a_ are also reflected in δ^13^C of C_4_ species. However, these results do not demonstrate whether genotypic differences in iWUE, which are expected to be much smaller than changes in response to water deficit, are predictable from screening for δ^13^C.

For different genotypes of maize, Monneveux et al. (2007) demonstrated that drought tolerant hybrids and inbreds showed lower δ^13^C values as well as higher grain yield under drought compared to drought susceptible inbreds. They also found a negative correlation between δ^13^C and ear dry weight at female flowering under drought conditions for the inbred lines contrasting for drought tolerance. Within the sample of drought-tolerant hybrids, however, no correlation of δ^13^C and yield under drought was found, which is likely due to the low variation in δ^13^C and drought tolerance between the selected genotypes.

Experimental evidence of a correlation of δ^13^C with WUE in C_4_ plants over different genotypes in well-watered conditions has been reported for *Setaria*, maize and sorghum. The most direct indication of a connection of δ^13^C and WUE in C_4_ species comes from QTL mapping in the interspecific *Setaria* RIL population (Ellsworth et al. 2020). The three QTL identified to control δ^13^C overlapped with QTL for WUE, leaf composition, biomass and transpiration, strengthening the hypothesis that there is a genetic link between δ^13^C and WUE. Moreover, a negative phenotypic correlation between δ^13^C and WUE of -0.51 was found in the well-watered treatment. The authors concluded based on the strong allelic effect on the relationship between δ^13^C and WUE that δ^13^C might be used as a proxy for WUE in C_4_ species in both well-watered and water limited conditions. Evidence for a genetic link between δ^13^C and WUE has also been shown in maize. Building on the QTL mapping by Gresset et al. (2014), Avramova et al. (2019) showed that a QTL for δ^13^C on chromosome 7 also influences WUE. An introgression from the drought susceptible donor parent in this region causes a decrease in WUE_plant_ and iWUE and an increase in grain δ^13^C, most likely by increasing stomatal conductance. The well-defined genetic material in this study also provided the framework to identify suitable molecular markers for selection of alleles affecting δ^13^C.

Further supporting evidence for a link between δ^13^C and WUE from experimental studies comes from weak, but significant phenotypic correlations of the two traits in 30 sorghum lines grown in the greenhouse as well as over individual plants of four lines grown in the field (Henderson et al. 1998). In this study, eight lines were selected for further investigation of C_i_/C_a_ and leakiness by combined measurements of gas exchange and on-line Δ^13^C. While no significant differences were detected in leakiness, there were significant differences in C_i_/C_a_ between the lines. In combination with the negative correlation of δ^13^C and WUE over the 30 lines this suggests that C_i_/C_a_ and thus iWUE was the main driver of δ^13^C variation. Contrastingly, Hammer et al. (1997) found no correlation between δ^13^C and WUE in 45 diverse sorghum lines, which they attribute to potential variation in respiration, non-stomatal water loss or leakiness due to the high diversity of the material.

Intraspecific variation in leakiness might be responsible for the sometimes weak correlations between δ^13^C and WUE. Only a limited number of studies have investigated variation in leakiness across genotypes of the same species. As leakiness cannot be measured directly, it is commonly derived from combined measurements of C_i_/C_a_ and Δ^13^C, using the model given in Eq. (3) (Henderson et al. 1992). The model relies on strong assumptions regarding energy production and consumption, fractionation factors and conductances of bundle sheath and mesophyll cells (Kromdijk et al. 2014). Due to the additional factors affecting dry matter δ^13^C that can lead to discrepancies in the relationship with short-term measurements of C_i_/C_a_, dry matter derived estimations are considered to be inaccurate representations of leakiness (Cousins et al. 2008). The only study we are aware of that used on-line measurements to investigate intraspecific differences in leakiness is the one by Henderson et al. (1998), in which no significant genotypic variation in leakiness of 30 sorghum lines was found.

The majority of leakiness studies focused on its responsiveness to environmental conditions to identify possible inefficiencies during the plant’s adaptation processes (Kromdijk et al. 2014). Changes of leakiness in response to environmental conditions, especially water deficit, can influence the correlation between Δ^13^C and WUE, as theoretically sign and magnitude of the correlation depends on leakiness (Eq. 3, Farquhar 1983). Henderson et al. (1992) demonstrated that leakiness is relatively stable over a range of temperatures, CO_2_ concentrations, and light intensities and in a recent study on sorghum no changes in leakiness were found in response to water deficit (Sonawane and Cousins 2020). Contrastingly, a significant response of leakiness to a high vapor pressure deficit has been observed for the C_4_ grass *Cleistogenes squarrosa* by Gong et al. (2017). The uncertainties in models used for calculating leakiness can have a large impact on its absolute values and its responses to environmental conditions (Kromdijk et al. 2014; Ubierna et al. 2018a), but the finely orchestrated coordination between PEPC and Rubisco as well as flexibility in the photosynthetic biochemistry has been proposed to constrain variations in leakiness (Bellasio and Griffiths 2014; Sun et al. 2012; Ubierna et al. 2013).

Overall, the sensitivity of detecting differences in WUE based on δ^13^C seems to be limited by the relatively small variation in δ^13^C with changes in C_i_/C_a_ in C_4_ plants, but reports of significant genetic and phenotypic correlations between δ^13^C and WUE indicate that at least major differences in WUE should be detectable through screening for δ^13^C. While variation in leakiness could lower the extent to which δ^13^C reflects differences in C_i_/C_a_, it did not cancel the correlation of WUE with δ^13^C for the majority of the studies reviewed.

## Conclusions

Using δ^13^C as an indirect trait to screen for WUE could facilitate the development of more water use efficient plants as one of the major challenges in breeding for climate resilience. While the relationship between δ^13^C and WUE in C_4_ crops is still less established than in C_3_ plants, evidence for a negative correlation of δ^13^C and WUE in C_4_ crops exists in the physiological as well as genetic context. Recent studies demonstrating the colocalization of δ^13^C and WUE QTL have delivered encouraging insights that it might be possible to identify plants with differential WUE through screening for δ^13^C. Additionally, these genetic studies greatly advance the possibilities for the identification of genes and molecular markers suitable for selection to improve WUE. Since intraspecific differences in δ^13^C and the correlation with WUE are less pronounced in C_4_ plants, it is likely that the sensitivity to detect differences in C_i_/C_a_ is lower than in C_3_ plants, but pronounced differences should still be reflected in δ^13^C and allow for pre-screening of suitable genotypes. More research is needed for investigating the effect of intraspecific variation in leakiness and post-photosynthetic fractionations. Unraveling the factors influencing δ^13^C at the physiological and genetic level in a variety of agronomically important crops will elucidate the contribution of different physiological and genetic factors to the expression of δ^13^C and estimate the extent to which it reflects WUE. With a profound knowledge of the underlying genetic mechanisms, δ^13^C can assist research and breeding efforts directed at improving WUE in the context of breeding climate resilient crops.

## Glossary

**C**_**i**_**/C**_**a**_: The ratio of intercellular to ambient CO_2_ partial pressure, determined by CO_2_ assimilation rate and stomatal conductance, assessed by gas exchange measurements of the plant leaf.
**Isotopic fractionation:**: Alteration in the stable carbon isotope ratio (^13^C/^12^C), occurring as a result of physical or biochemical processes during the transport and metabolism of carbon in the plant.
**Carbon isotope discrimination (Δ**^**13**^**C):**: The preferential assimilation of the lighter stable carbon isotope ^12^C over the heavier ^13^C during the process of photosynthesis in plants. Δ^13^C is calculated as the difference between the δ^13^C of the analyzed plant sample (δ^13^C_p_ typically plant dry matter) and δ^13^C_air_ surrounding the plant (Farquhar et al. 1982).
**Carbon isotopic composition/signature (δ**^**13**^**C):**: The stable carbon isotopic composition of a sample, e.g. air or plant material (δ^13^C), expressed as the ^13^C/^12^C ratio of the sample (R_s_) in reference to the ^13^C/^12^C ratio of the Pee Dee Belemnite Standard (R_PDB_), a fossil with an exceptionally high amount of the ^13^C isotope (Farquhar et al. 1982). More negative values for δ^13^C indicate a high discrimination against ^13^C. δ^13^C is successfully used as an indirect trait for screening for improved water use efficiency in C_3_ plants (Condon et al. 2004).
**Intrinsic water use efficiency (iWUE):**: The ratio of CO_2_ assimilation rate to stomatal conductance, measured at the leaf level of the plant by means of infrared gas analyzers.
**Whole plant water use efficiency (WUE**_**plant**_**):**: The ratio of the whole plant biomass to the total volume of water transpired by the plant.
**Water use efficiency (WUE):**: The ratio of yield (grain or biomass) to water received or evapotranspired by the system (e.g. field plot Ellsworth and Cousins 2016).
**Vapor pressure deficit (VPD):**: The difference between the amount of moisture in the air and the maximum air moisture at saturation.
